# Analysis and design of InAs nanowire array based ultra broadband perfect absorber

**DOI:** 10.1039/d1ra06812a

**Published:** 2021-11-23

**Authors:** Mohammad Muntasir Hassan, Fariba Islam, Md Zunaid Baten, Samia Subrina

**Affiliations:** Department of Electrical and Electronic Engineering, Bangladesh University of Engineering and Technology Bangladesh samiasubrina@eee.buet.ac.bd; Institute of Information and Communication Technology, Bangladesh University of Engineering and Technology Bangladesh hmuntasir@iict.buet.ac.bd

## Abstract

An ultra-broadband perfect absorber has a wide range of applications which include solar energy harvesting, imaging, photodetection *etc.* In this regard, InAs nanowire (NW) based structure is investigated in this work for achieving an ultra broadband perfect absorber. Finite difference time domain (FDTD) based numerical analysis has been performed to optimize the InAs nanowire based structure to obtain an efficient light absorber by varying different dimensional parameters. Mie theory and guided mode resonance based theoretical analysis is developed to validate the results and to get an insight into the tunability of the nanowire based structure. Moreover, the theoretical analysis elucidates the underlying physics of light absorption in nanowires. To achieve ultra broadband absorption, multi radii InAs nanowire based arrays are investigated and it is found that they exhibit superior performance compared to single radius NW based structures. The computed light absorption efficiency (LAE) and short circuit current density values are enhanced to 97% and 40.15 mA cm^−2^ at 10° angle of incidence for the optimized quad radii NW array within the wavelength range of 300 nm to 1000 nm and 300 nm to 1200 nm, respectively. Moreover, the absorption spectra for these structures are polarization independent and exhibit robust performance for varying angle of incidence. In addition, arrangement of the NW array (hexagonal or square) has negligible effect on the absorption spectra. Such ultra-broadband absorption capability of the proposed structure compared to existing works suggests that the InAs nanowire based structure is very promising as light absorber with prospects in the fields of photo detection, solar power generation, perfect cloaking, photochemistry and other thin film photonic devices.

## Abstract 2

Mie theory and GMR based theoretical framework support the numerical results that resonant wavelength increases with increasing InAs NW diameter. By employing NWs of different diameters in a single array, an ultra-broadband perfect absorber has been achieved.

## Graphical abstract

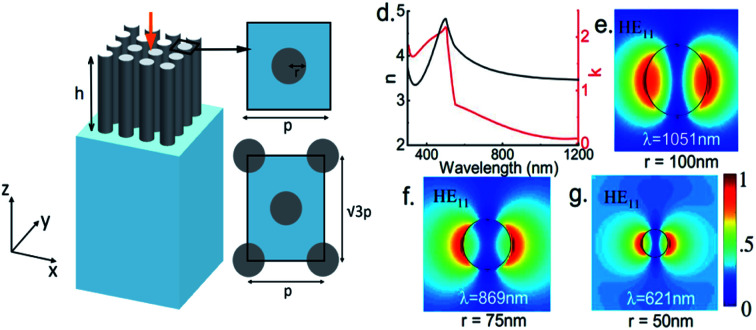

## 1 Introduction

Ultra broadband perfect light absorbers has been a research attraction in recent times because of its vast field of application like solar cells,^1^ imaging,^2^ photo detection,^3^ shielding,^4^ sensing,^5^ optical data storage *etc.*^6^ Narrow band absorption is primarily required for sensing whereas wideband absorption is needed for energy harvesting^7^ applications. To obtain ultra broadband near perfect absorbers researchers have employed a number of novel structures and materials including metamaterials,^8–10^ nanowires^11^ and nanopillars.^12^ Different novel nano-photonic structures, such as three-dimensional photonic crystals,^13^ triangular and pyramid gratings,^14^ nanocones,^15^ nanowires,^16,17^ plasmonic nanostructures,^8,18,19^ nanoholes^20,21^*etc.* have already been theoretically proposed and experimentally demonstrated for light-trapping in solar cells and photodiodes.^22^ Most of these works are based on Si or GaAs as these materials are widely used and fabrication technology is well established. The main goal of all the novel structures is to achieve improved absorption efficiency and in this regard nanowires (NWs) can be a promising candidate. Till date no work has been reported on ultra broadband perfect absorber employing solely semiconductor nanowires (NWs). Nanowires are anisotropic structures with base dimensions of the order of nanometers and longitudinal dimensions of the order of few microns and this shape enables the absorption of light in a more efficient manner compared to an equivalent volume of planar material.^23^ While nanowires of any semiconducting material are extremely efficient for optoelectronic applications, III–V compound semiconductors are especially promising for photonic devices because of their direct band gaps.^23,24^ III–V nanowire (NW) arrays have been extensively used in a variety of configurations like core–shell,^25^ core-multi shell,^26^ inclined NW array^27^ for photo detection^3^ and photovoltaics^27–30^ for their phenomenal field confinement.

In this work, we focus on InAs nanowire array based highly efficient absorber as it has large absorption coefficient in comparison to other semiconducting materials like silicon, GaAs or InP^31^ and yet to be explored extensively for ultra broadband absorber applications. In addition, optical absorption wavelength range for InAs is large due to its lower bandgap. Researchers have worked on GaAs,^32–35^ silicon,^36–38^ and InP^25,28,39–45^ NW based solar absorbers but the work on ultra broadband light absorption using InAs NWs is very few. Wu *et al.*^46^ investigated the dependence of optical response on the InAs NW geometry and Rahman *et al.*^41^ reported wavelength selective absorption in InAs NWs. Kupec *et al.* worked on InP/InAs NW based solar cells and analyzed the absorption characteristics of NW arrays of various diameters.^40^ Though InAs nanowire fabrication is still challenging, wafer scale production of InAs nanowire has been reported.^47^ In the present study, we have proposed InAs nanowire (NW) based structure for ultra broadband absorption of light and analyzed the absorption characteristics using guided mode resonance and Mie theory based theoretical framework and finite difference time domain (FDTD) based numerical technique. Absorption characteristics of the NW based structure for both square and hexagonal unit cell can be tuned by varying the radius and filling ratio of the NWs and thus an optimized structure is found. To achieve ultra broadband absorption of light multi radii InAs NW based structure is proposed and is found to be the most efficient as it exhibits high short circuit current density and perfect light absorption for a broad range of wavelength which covers significant portion of the solar spectrum. Polarization and angle sensitivity of the optimized structure is also investigated and the results signify the potential of the proposed structure as the absorber layer of thin-film photonic devices and such absorption characteristics can have profound impact in the field of photochemistry^48^ and photoelectrochemistry^49^ as well. The analysis and discussions would also give an intuitive perception behind the ultra broadband absorption characteristics of the optimized structure.

## 2 Simulation framework and sparse single radius NW array

**Fig. 1:**
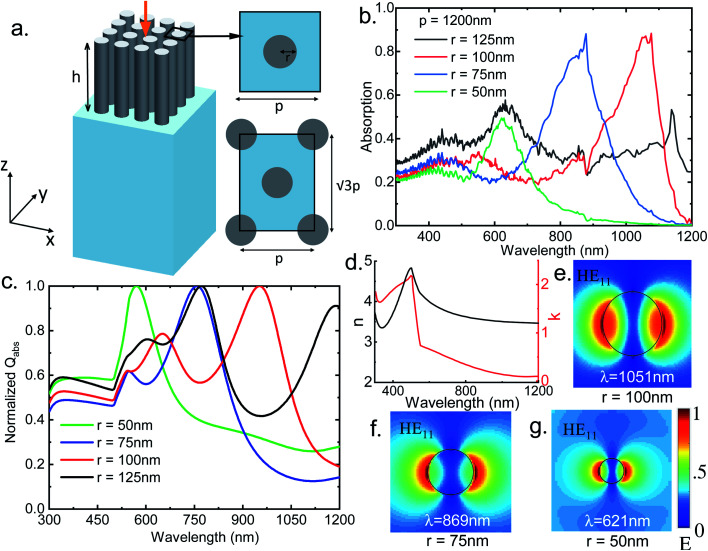
(a) Schematic of the designed InAs NW array based absorber structure (the orange arrow indicates the direction of the incident light); top view of square unit cell and hexagonal unit cell are shown as insets on the top and bottom respectively; (b) absorption spectra for sparse InAs NWs of varying radius with a period of 1200 nm for HE illumination; (c) calculated *Q*_abs_ values from Mie theory based theoretical framework for InAs NWs of varying radii; (d) refractive index (*n*) and extinction coefficient (*k*) values of InAs used in this study; (e)–(f) mode profiles of the 100 nm, 75 nm and 50 nm radius NW for the respective HE_11_ resonant modes.

The proposed structure consists of vertically aligned InAs nanowire (NW) array placed on the SiO_2_ substrate. Heights of the NWs and substrate are kept constant at 2 μm and 5 μm, respectively throughout the study. Light absorption efficiency of the proposed structures are analyzed by solving Maxwell's equations using three dimensional finite difference time domain (FDTD) based numerical analysis technique.^50^ The schematic of the proposed structure is shown in Fig. 1(a). Two different combinations of the NW arrays are analyzed where the NWs are arranged in square and hexagonal manner. Square and hexagonal unit cell with the black rectangles show the simulation boundary of the unit cells in the *xy* plane (inset of Fig. 1(a)). Radius (*r*) and height (*h*) of the NWs as well as period (*p*) of the unit cells are also shown as insets in Fig. 1(a). Periodic boundary conditions are used in the *x* and *y* direction and perfectly matched layer (PML) boundary conditions are used in the *z* direction. To consider the frequency dependent extinction coefficient of InAs and SiO_2_, experimentally reported extinction coefficient (*k*) and refractive index (*n*) values are used.^31^ Plane wave source within the wavelength range 300–1200 nm is used to illuminate the structure with TM like HE polarization and the direction of propagation is along the negative *z* direction as shown with an orange arrow in Fig. 1(a). Solar spectrum^51^ has the highest intensity in this wavelength range and ultra broadband perfect absorption of light within this range is desired for thin film photonic devices applications. Fitted *n* and *k* values of the InAs within this wavelength range is plotted in Fig. 1(d). Absorption of light in the NW array is calculated using the relation *A* = 1 − *R* − *T*, where *R* is the reflection from the NW array and *T* is the transmission through the structure.

To investigate the effect of nanowire radius on the absorption of light, absorption spectra of InAs NWs having radius from 50 nm to 125 nm are analyzed and plotted in Fig. 1(b). NWs are arranged in square unit cell and period of the unit cell is kept constant at 1200 nm. Here the volumetric filling ratio (FR) of the NWs vary from 0.55% to 3.4%. As the volumetric filling ratio (FR) of the nanowire is very small and the inter-NW spacing is large, the NWs can be considered sparsely arranged. Here filling ratio (FR) is defined as1
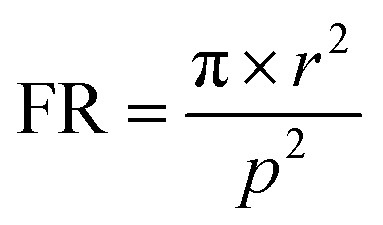
From Fig. 1(b), it can be seen that up to 500 nm wavelength absorption is almost same in NWs of different radii. However, in longer wavelength range, the effect of radius on the absorption is more conspicuous as the resonant absorption wavelength increases with increase in the NW radius. In lower wavelength range, intrinsic absorption property of the InAs contributes the absorption of light whereas in higher wavelength light trapping is caused by the resonant mode of the NWs.

Mie theory based theoretical analysis corroborates the absorption spectra of the sparse InAs NWs. According to the Mie theory, analytical expression of absorption efficiency *Q*_abs_ is given as^43,52,53^2
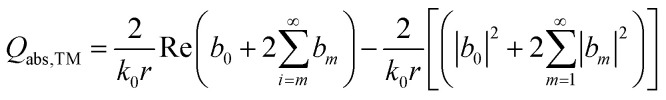
Here *b*_*i*_ are the Mie scattering coefficients for a plane wave incident on the infinite nanowires given by3
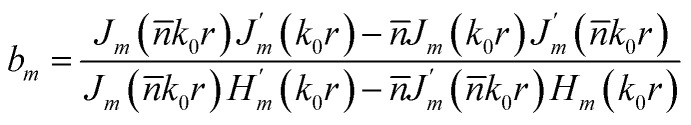
where *r* is the radius of the NW and *k*_0_ is the wave vector along the NW axis inside the NW, *J*_*m*_ and *H*_*m*_ are *m*th order Bessel and Hankel functions of the first kind respectively. *n̄* = *n* + i*k* is the complex refractive index of the NW. For varying radii of the NWs calculated *Q*_abs_ from Mie theory is plotted in Fig. 1(c) and it correlates well with the simulated absorption spectra shown in Fig. 1(b).

Light trapping in higher wavelengths can be further explained by guided mode resonance in the NWs. A semiconductor NW can be considered as cylindrical resonator where the light is trapped by multiple total internal reflections from the edge of the NWs. These resonant modes are guided modes where the NWs can be considered as the wave guides and can be described from the classical wave guide theory by solving Maxwell's equations with appropriate boundary conditions.^54^ Guided modes are essentially morphology-dependent resonances^55^ arising from the finite NW size and the large refractive index contrast of the NW with respect to its surroundings. It should thus be expected that any high-index semiconductor used in light trapping applications could benefit from these types of resonances. Guided modes are characterized by their azimuthal mode number (*m*) and radial mode number (*n*) and for normal incidence only hybrid TM-like HE_1*n*_ modes are effectively excited in the NW due to the symmetry of the incident light.^36,45^ Assuming that the real part of the propagation constant of the mode along NW axial direction approaches zero, the resonant wavelength can be approximated by^42,43,56^4
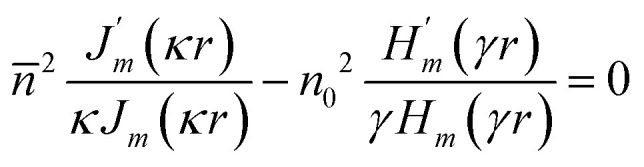
Here *γ* and *κ* are transverse wave vectors inside the NW having refractive index *n̄* and in air (*n*_0_ = 1) respectively. Using eqn (4) fundamental HE_11_ resonant modes can be calculated and the absorption property can be tuned by varying the radius of the NW. For InAs NWs, the calculated HE_11_ resonant wavelengths using eqn (4) are shown in Table 1 and it is found that these values match well with the simulated simulated peak absorption wavelengths. The trend also matches with the theoretical results obtained from Mie theory. From Fig. 1(b) it is found that InAs NWs having radius 50 nm, 75 nm and 100 nm exhibits highest absorption in these HE_11_ resonant modes. Poor absorption for NW with 125 nm radius can be explained from the guided mode theory as the calculated HE_11_ resonant mode for 125 nm radius NW is outside the simulated wavelength range. The mode profiles at *λ* = 1051 nm, 869 nm and 621 nm for NWs having radii 100 nm, 75 nm and 50 nm respectively shown in Fig. 1(e)–(g) support the existence of HE_11_ resonant modes. It is noteworthy that Mie theory and guided mode resonance based eqn (4) is derived for infinitely long NWs having large lattice periods where modes of a single nanowire is unperturbed. This can explain the slight mismatch between the calculated and observed values in Table 1.

**Table 1:** Comparison of calculated and simulated resonant wavelengths for sparse InAs NWs

Radius (*r*)	*m*	*n*	Calculated *λ* (nm)	Calculated *λ* (nm)	Numerical simulation *λ* (nm)
			From eqn (4)	Mie theory	
125 nm	1	1	1276	1190	Outside simulated spectral range
100 nm	1	1	1002	955	1051
75 nm	1	1	868	760	869
50 nm	1	1	601	567	621

When the nanowires are more closely packed with smaller periods, the resonant modes of one NW gets coupled to the neighbouring NWs as a result we can observe a high absorption throughout the complete wavelength range which is discussed in the following sections.

## 3 Single radius InAs nanowire arrays

**Fig. 2:**
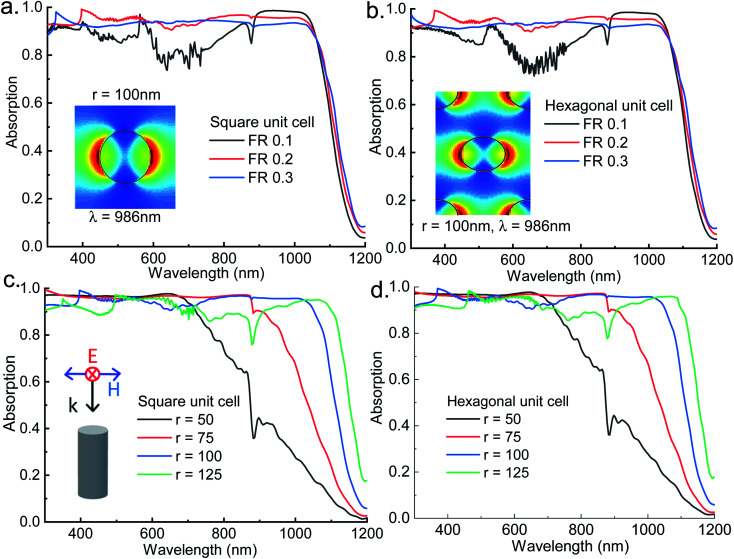
Absorption spectra of (a) square and (b) hexagonal unit cell InAs NW arrays for varying fill factor by keeping the radius of the NW constant at 100 nm, inset of (a) shows the mode profile for *λ* = 986 nm of square unit cell with filling ratio 0.2, inset of (b) shows the mode profiles of *λ* = 986 nm for filling ratio 0.2 for hexagonal unit cell; absorption spectra of (c) square and (d) hexagonal unit cell InAs NW arrays for varying radius of the NW by keeping the FR constant at 0.2; polarization and orientation of the incident light is also shown as inset in (c).

From the analysis of sparse NW arrays it has been found that absorption of light below band gap energy is governed by the presence of guided resonant modes in NWs. To investigate the same for closely spaced nanowire arrays, squarely arranged InAs NWs having 100 nm radius are simulated for filling ratios of 0.1, 0.2 and 0.3. From the simulated absorption spectra shown in Fig. 2(a) it is observed that broadband absorption of light occurs and absorption increases significantly compared to sparse NW arrays. As the NW spacing decreases the modes of the NWs are perturbed by the neighboring NWs and broadband light absorption is achieved. Mode profile for HE_11_ mode of 100 nm InAs NW with FR 0.2 at *λ* = 986 nm is shown in Fig. 2(a) (inset) which supports the presence of guided mode resonance. It is evident that as the nanowire spacing is decreased, a blue shift in HE_11_ resonant mode has occurred as the resonant mode has shifted from 1051 (Fig. 1(e)) nm to 986 nm (Fig. 2(a) inset). The blue shift occurs due to the coupling of the near field evanescent waves with neighboring NWs as the spacing decreases. The stronger coupling of the evanescent fields increases the peak absorption as well enabling broadband absorption of light.^41^ At the same time, the amount of absorbing InAs material in these cases is significantly higher compared to sparse NW arrays. However, as the filling ratio increases over 0.2, absorption decreases due to the increased reflection from the NWs which can be corroborated from the Fresnel's reflection formula by calculating the effective refractive index of the NW layer using effective medium theory.^57^ Similar analysis is performed for hexagonally arranged InAs NW arrays having 100 nm radius with different filling ratios of 0.1, 0.2 and 0.3 and results are shown in Fig. 2(b). The FR of hexagonal unit cell is calculated using the relation5
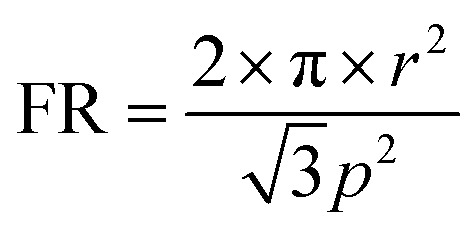


It is evident that arrangements of NW arrays have negligible effect on the absorption as the results for hexagonal unit cell closely matches with the results of square unit cell. This result is expected as the guided mode resonance is independent of the nature of the unit cell of the NWs. The mode profile at *λ* = 986 nm for hexagonal unit cell of 100 nm radius InAs NW with 0.2 FR shown in Fig. 2(b) (inset) corroborates this claim.

Next to find out the effect of the radius of the NW on the absorption profile for closely packed NWs, radii of the InAs NWs were varied keeping the FR constant at 0.2 for both square and hexagonal unit cell and the results are shown in Fig. 2(c) and (d) respectively. The results for both square and hexagonal unit cell match closely as expected and they correlate with the results found in Fig. 1(c). The absorption wavelength range increases as the radius of the NW increases and cut off wavelength of the absorption range depends on the HE_11_ resonant mode which increases with radius as shown in Table 1. Though the absorption wavelength range for squarely arranged 125 nm NW array is higher but overall absorption within the 300 nm to 1200 nm wavelength range is higher for 100 nm NW array. From the above analysis it is found that 100 nm radius NW array with 0.2 FR is the optimized structure for single radius InAs NW array that enables broadband light absorption.

**Fig. 3:**
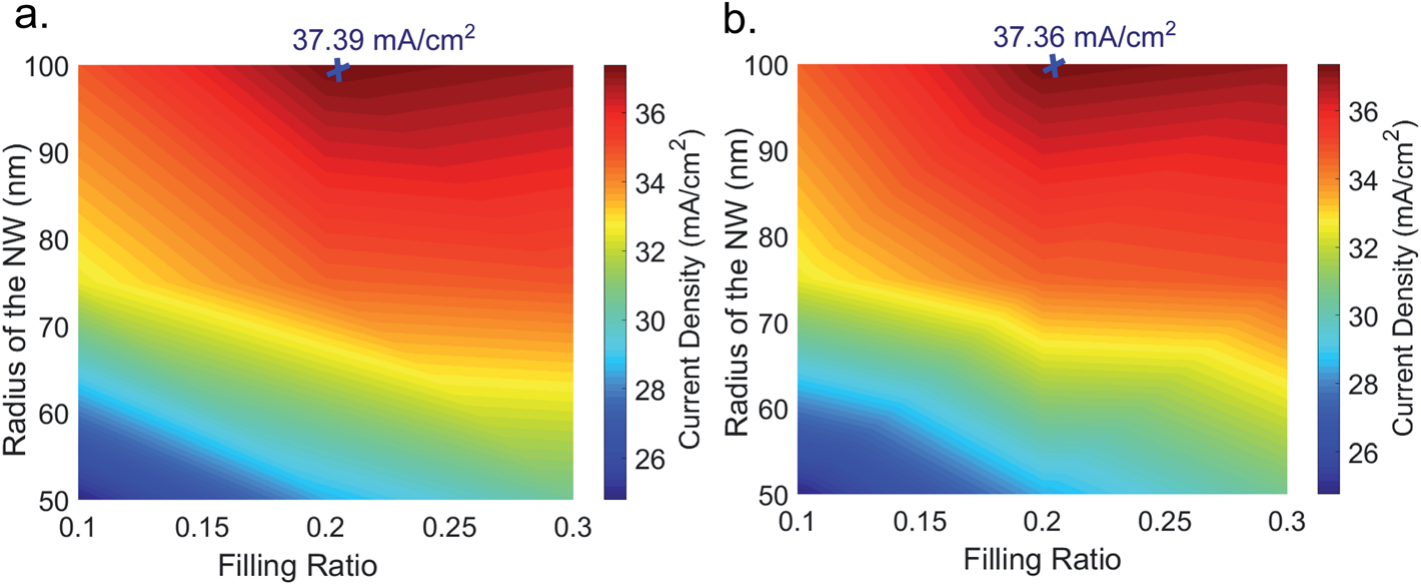
False color plot of calculated short circuit current density for different combinations of radius and filling ratio of NW for (a) square unit cell and (b) hexagonal unit cell of InAs NW structure.

We calculated the short circuit current density values from the absorption spectra of both the unit cells for various NW radii and filling ratios. The short circuit current density is calculated using the following formula6
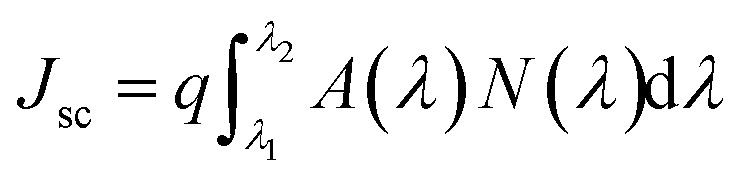
where *A*(*λ*)is the absorption data, *N*(*λ*) is the number of photons per unit area per unit second from the standard solar AM 1.5D spectrum,^51^*λ*_1_ = 300 nm and *λ*_2_ = 1200 nm. Calculated current density values for square and hexagonal unit cell of InAs NW are shown in Fig. 3(a) and (d) respectively. The plot shows that the maximum current density is found for a radius of 100 nm and filling ratio of 0.2. The computed maximum current density for square unit cell and hexagonal unit cell is 37.39 mA cm^−2^ and 37.36 mA cm^−2^ respectively. Current density values for 125 nm radius NW with 0.2 filling ratio are calculated as well and found to be 37.21 mA cm^−2^ and 37.32 mA cm^−2^ for square and hexagonal unit cells, respectively. These values confirm that InAs NW array with 100 nm radius and 0.2 filling ratio is the optimized light absorbing structure for single radius case. Since both the unit cells give almost similar results and it is easier to fabricate and computationally less expensive to simulate square unit cell so we have considered square unit cell for further study.

## 4 Multiple radii nanowire arrays

Broadband absorption spectra and the high short circuit current density have been achieved by optimizing the single radius InAs NW array structure. However, the absorption spectra can be further optimized to make it ultra broadband perfect absorber by employing multi radii InAs NW array. In Fig. 1(c) it is shown that resonant absorption modes of the NWs depend on the radii of the NWs and by tuning the radii of the NWs, absorption within the desired wavelength range can be achieved. This observation has inspired the investigation of multi radii NW array as nanowires with different radii will confine light of different wavelengths enabling ultra broadband absorption of light.

**Fig. 4:**
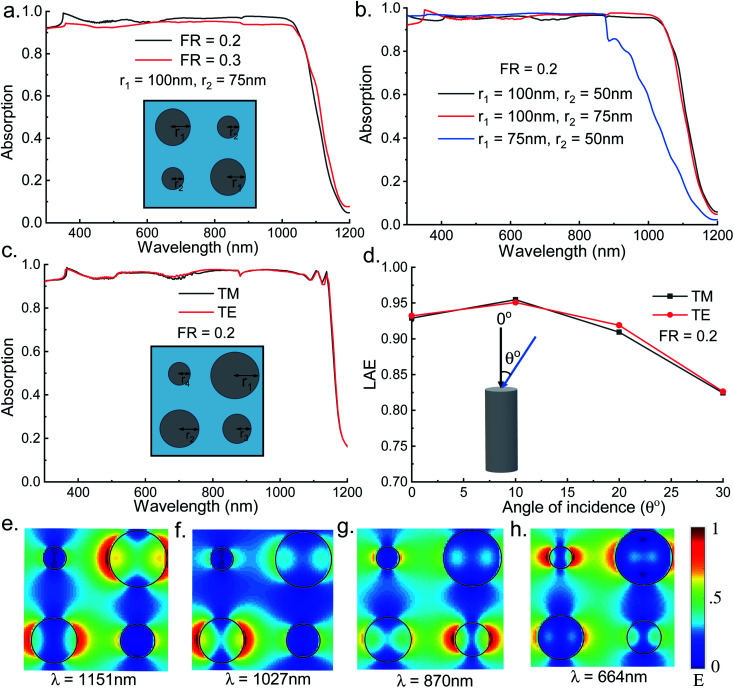
(a) Absorption spectra of double radii NW structure for FR of 0.2 and 0.3 for TM illumination, inset showing the top view of the unit cell; (b) absorption spectra of double radii NW structure for various combinations of the NWs; (c) absorption spectra of quad radii NW structure for FR of 0.2 for both TM and TE polarized light, inset shows the top view of the unit cell; (d) calculated LAE values from the absorption spectra of quad radii NW structure for varying angle of incidence for both TM and TE polarized light; (e)–(h) mode profiles at different wavelengths for the quad radii InAs NW unit cell for TM polarization.

Multiple radii NW arrays are investigated in two different configurations. In Fig. 4(a) (inset) we see the top view of the unit cell of the structure with double radii NWs where the diagonal NWs have the same radii which are shown as *r*_1_ and *r*_2_. To analyze the effect of FR on the absorption for double radii structure unit cells with 100 nm and 75 nm, radii nanowires are simulated with FR of 0.2 and 0.3. The FR for double radii NW structure is calculated using the following relation7
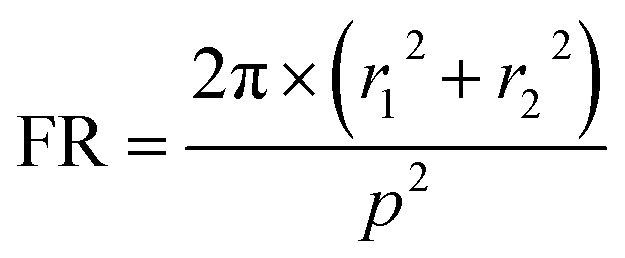


Fig. 4(a) shows that the structure having 0.2 FR gives slightly better result than the structure having FR 0.3 and the absorption is higher than the optimized single radius NW structure. Next for a filling ratio of 0.2, we have computed the absorption spectra of the structure for three different combinations of the radii- 75 nm and 50 nm, 100 nm and 75 nm, and 100 nm and 50 nm. The results are plotted in Fig. 4(b) and the effect of radius of the NW is clearly distinguishable as the 75 nm and 50 nm double radii structure has low absorption bandwidth than the other counterparts. The highest absorbing double radii NW structure is found for the combination of 100 nm and 75 nm radius with FR of 0.2. From the results and analysis, it is evident that radius of the NW governs the absorption wavelength range while the FR determines the amount of light absorbed.

Next quad radii InAs NW array with four different radii NWs is simulated to achieve ultra broadband application. The unit cell for this structure is shown as in inset of Fig. 4(c). Here InAs nanowires of four different radii 50 nm, 75 nm, 100 nm and 125 nm are utilized and the calculated guided resonant modes lie within the wavelength range of 621 nm to 1250 nm. As four different radii NWs have resonant modes in different wavelengths so it would enable ultra broadband absorption of light. Like single and double radii nanowire structure, the absorption spectra for this structure is calculated for filling ratio of 0.2. The FR for quad radii NW structure is calculated by modifying eqn (6)8
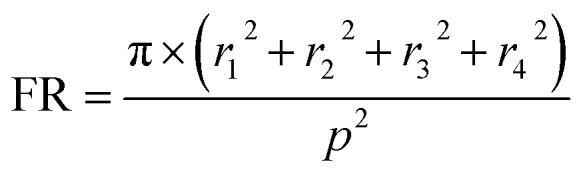
The result is plotted for both TM and TE polarized light for normal incidence in Fig. 4(c) and from this graph it is evident that quad radii NW structure absorbs light of even higher wavelength as expected and it shows enhanced light absorption efficiency for both TM and TE polarized light. Moreover, in order to investigate the angle of incidence (AOI) dependency of ultra broadband absorption of the quad radii nanowire structure, the angle of incidence is varied for both TM and TE polarization. The angle of incidence, *θ*, is defined as the variation from the normal incidence as shown in the inset of Fig. 4(d). The light absorption efficiency (LAE) is calculated from the absorption data of different angle of incidence using the following formula9
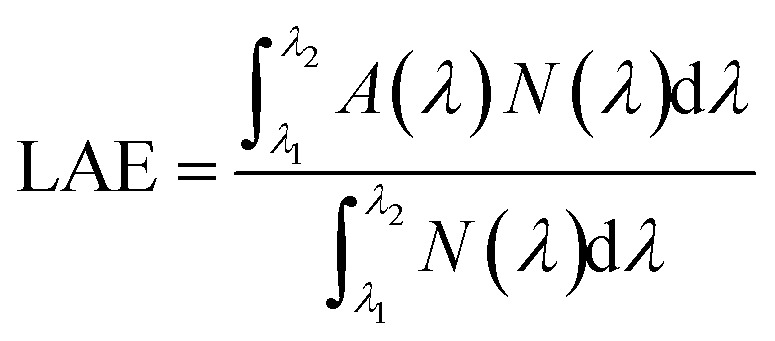


Light absorption efficiency tells us how much of the incident light is being absorbed by the structure and here *A*(*λ*) is the absorption data, *N*(*λ*) is the number of photons per unit area per unit second from the standard solar AM 1.5 spectrum.^51^ The LAE for 300 nm to 1200 nm wavelength range for different angle of incidence for both TM and TE polarization are shown in Fig. 4(d) and it is found that absorption actually increases for 10° angle of incidence and decreases again for further increase in angle of incidence. The LAE for 10° angle of incidence is very high and calculated to be 95.46%. Moreover, the structure is found to be polarization independent and thus enhances its credential for photovoltaic applications. Alternatively, we can suggest that a structure with quad radii NWs grown with an inclination of 10° will absorb light more efficiently for normal incidence. The broadband absorption of light in quad radii NW array can be explained from the mode profiles shown in Fig. 4(e–h). It is evident from these mode profiles that light with lower wavelengths gets coupled with NWs of smaller radii. This is in correlation with the results from the analysis of guided resonant modes and Mie theory based theoretical framework. In quad radii NW array, by employing NWs of varying radii light with different wavelengths can be trapped within the array, thus enabling the ultra broadband perfect absorption of light.

To compare different InAs NW based structures discussed above three different figure of merits are used-short circuit current density (*J*_SC_), light absorption efficiency (LAE) and enhancement factor (EF). LAE for different structures were calculated for two different wavelength ranges – 300 nm to 1200 nm and 300 nm to 1000 nm. Enhancement factor is defined as10
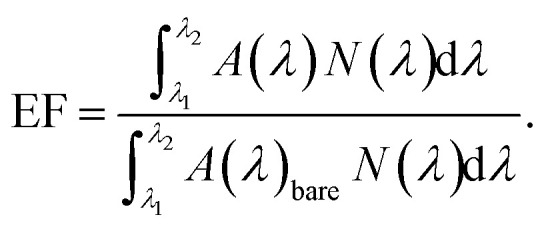
Here *A*(*λ*)_bare_ is the wavelength dependent absorption in a bare 400 nm thick film of InAs that has a volume equal to that of the nanowire. Short circuit current density, LAE and EF for different optimized InAs NW based structures are summarized in Table 2. The highest short circuit current density for normal incidence is found to be 39.08 mA cm^−2^ for quad radii InAs NW structure which is significantly higher than GaAs NW^17^ and InP NW^43^ based structure. If the quad radii InAs NW structure is illuminated at 10° angle of incidence, the short circuit current density value soars even higher at 40.15 mA cm^−2^ accounting for ∼95% conversion efficiency comparing with the AM 1.5 spectrum^51^ (∼42 mA cm^−2^). Moreover, for this configuration this structure has phenomenal light absorption efficiency of 97% within the wavelength range 300 nm to 1000 nm. For normal incidence the highest light absorption efficiency of 96.31% was found for 100 nm and 75 nm double radii InAs NW structure with FR 0.2 within the wavelength range 300 nm to 1000 nm. Table 3 shows the comparison of the results from the present study with the previous works using semiconductor nanowire based absorber and from this table it is evident that this work produces significantly better result in terms of *J*_SC_ as well as enables ultra broadband perfect absorption. In addition, higher light absorption efficiency and enhancement factor values suggest that InAs NWs can be used as the absorber layer in thin film photonic devices which can increase their efficiency significantly.

**Table 2:** Comparison of calculated short circuit current density, light absorption efficiency and enhancement factors for different configurations of InAs based nanowire structures

Structure	*J*_SC_ (mA cm^−2^)	LAE (%)	LAE (%)	EF
	300–1200 nm	300–1200 nm	300–1000 nm	300–1200 nm
Bare 400 nm thick InAs	23.78	56.55	60.72	1
100 nm rad 0.2 FR (square)	37.39	88.89	94.74	1.57
100 nm rad 0.2 FR (hexagonal)	37.36	88.82	94.69	1.57
100 nm and 75 nm double rad 0.2 FR	37.73	89.7	96.31	1.59
Quad radii 0.2 FR	39.08	92.9	95.78	1.64
Quad radii 0.2 FR with 10° AOI	40.15	95.46	97.0	1.69

**Table 3:** Comparison of calculated short circuit current density of InAs nanowire based structure with existing semiconductor nanowire based structures

Structure type	Wavelength range (nm)	*J*_SC_ (mA cm^−2^)	Ref.
Si NW	300–900	14.5	52
Inclined Si NW	310–1127	32.39	58
InP NW	300–1000	33.13	43
GaAs NW	300–1000	29.88	17
GaAs NW	400–1100	30	59
GaAs conical NW	400–860	28	60
InAs NW	300–1200	40.15	This work

## 5 Conclusions

In this study, we have optimally designed single radius and multiple radii InAs NW arrays to achieve ultra broadband perfect absorption that can be used as the active layer of thin film photonic devices. Geometrical dimensions for vertically aligned single, double, and multiple diameters of InAs NW arrays are numerically investigated using FDTD simulations. The absorption spectra of the proposed structure are theoretically calculated using Mie theory and guided mode resonance and the obtained values exhibit good agreement with the simulation results. To achieve ultra broadband perfect absorber multi radii optimized NWs structure is employed with filling ratio 0.2 and the highest short circuit current density of 40.15 mA cm^−2^ and LAE of 97% are obtained for 10° angle of incidence within the wavelength range of 300 nm to 1000 nm and 300 nm to 1200 nm respectively. Moreover, both the hexagonal and square unit cells show closely matched absorption spectra. The optimized structure is found to be polarization insensitive and the LAE remains above ∼85% up to 30° angle of incidence whereas for normal incidence of light it becomes 95.78%. In addition, the LAE can be as high as 96.31% for normal incidence of light using double radii nanowire array. The analysis suggests that optimum amount of absorbing material *i.e.* filling ratio is required for the highest absorption of light while the absorption wavelength range is determined by the radius of the NWs. This is the first reported semiconductor NW based ultra broadband perfect absorber and it can pave the way for efficient thin film photonic devices by offering design-flexibility and reducing fabrication and processing complexity of such single layer InAs nanowire structures.

## Author contributions

Mohammad Muntasir Hassan: conceptualization, data curation, formal analysis, visualization, software, writing – original draft, writing – review & editing; Fariba Islam: conceptualization, data curation, formal analysis, software, writing – original draft; Md Zunaid Baten: co-supervision, writing – review & editing; Samia Subrina: conceptualization, writing – review & editing and supervision.

## Conflicts of interest

There are no conflicts to declare.

## Supplementary Material
